# The Impact of Mobile Apps on Alcohol Use Disorder: A Systematic Review Protocol

**DOI:** 10.2196/resprot.6975

**Published:** 2017-04-04

**Authors:** Antoine SA Sawares, Nelson Shen, Yunlin Xue, Alexxa Abi-Jaoude, David Wiljer

**Affiliations:** ^1^ Centre for Addiction and Mental Health Toronto, ON Canada; ^2^ University of Toronto Faculty of Medicine Toronto, ON Canada; ^3^ University of Toronto Institute of Health Policy, Management and Evaluation Toronto, ON Canada; ^4^ University Health Network Toronto, ON Canada

**Keywords:** mobile health, apps, alcohol use disorder, systematic review, protocol, mental health, addiction, alcoholism

## Abstract

**Background:**

Alcohol use disorder (AUD) is among the most prevalent mental disorders worldwide and is associated with a diverse range of physical and psychological comorbidities. Despite various types of treatment, there are many barriers to accessing treatment (ie, stigma, cost, accessibility of service, etc). Mobile apps have the potential to overcome these barriers and provide support to those who need it.

**Objective:**

The purpose of this systematic review is to assess the effectiveness of mobile apps in reducing alcohol consumption for individuals with AUD and understand the psychological outcomes of using the apps (ie, client empowerment, self-efficacy, etc).

**Methods:**

The search strategy was applied to 7 health sciences and interdisciplinary databases. Two reviewers will independently assess all titles and abstracts for relevance and then full texts of relevant articles for eligibility. To be included, the article must be a quantitative evaluation of clinical outcomes using the intervention and the intervention must be a consumer-facing app focused on supporting individuals with AUD. Two reviewers will independently extract data from all eligible articles using a standardized extraction worksheet and will independently assess the study quality. A meta-analysis will be conducted if appropriate. Depending on outcomes reported, pooled risk ratios or standardized mean differences will be calculated and reported in the review.

**Results:**

The search strategy yielded 699 unique citations. Of those, 63 (9.0%, 63/699) articles were assessed as relevant for full-text review. The full-text reviews are currently underway and the final review is projected to be completed in the summer of 2017.

**Conclusions:**

There is potential for mobile apps to support individuals with AUD to reduce their alcohol consumption. This review will be the first to assess the effectiveness of AUD mobile apps and client experiences using the apps.

## Introduction

In many cultures, the consumption of alcohol is considered a social norm. While there are beneficial effects of moderate consumption, including decreased risk for heart disease, ischemic stroke, and diabetes [1], research has shown many risks associated with alcohol use linked to heavy drinking patterns [2]. In 2010, the global per capita consumption of alcohol (ages 15 and up) was equivalent to 6.2 liters of pure alcohol consumed per calendar year. Furthermore, 16.0% of those drinkers engaged in heavy episodic drinking [3]. Alcohol-related harm is determined by the volume of alcohol consumed, the pattern of drinking, and, on rare occasions, the quality of alcohol consumed. According to the Diagnostic and Statistical Manual of Mental Disorders, 5th edition (DSM-5) alcohol use disorder (AUD) is a problematic pattern of alcohol use defined by specific diagnostic criteria including a cluster of behavioral and physical symptoms leading to clinically significant impairment or distress. Previous DSM editions defined problematic alcohol use through two distinct categories: abuse and dependence [4].

AUD is among the most prevalent mental disorders worldwide and contributes substantially to global morbidity and mortality [5,6]. The World Health Organization estimated that over three million deaths per year are linked to AUD—slightly more than lung cancer and HIV/AIDS mortalities combined [3]. Alcohol use has been associated with diverse physical and psychiatric comorbidities. It is affiliated with over 200 different diseases, conditions, and injuries [7]. Among those who drink heavily, women tend to experience more heart, liver, and brain damage from alcohol compared to men [8]. In addition to disease risk, alcohol consumption could also lead to financial burdens for the drinker and harm others around them [2].

Over the years, numerous therapeutics for alcohol management have been developed. The interventions range from various types of counseling, for example, brief intervention [9] or motivational interviewing [10], to using anti-craving medication (eg, Naltrexone and Acamprosate) [11]. While counseling primarily targets behavioral changes, pharmacological agents alter physiological responses to alcohol intake. Despite the various types of treatments available, there are many barriers to accessing treatment. For non-treatment seekers, stigma and cost of treatment are reported to be the main barriers. For treatment seekers, stigma is a significant barrier. In addition, barriers to treatment include long wait-times and a limited number of trained personnel [12]. Brief intervention is a treatment that faces this limitation. Potential issues regarding pharmacotherapy include side effects, lack of continuous monitoring, and potentially low patient compliance [13].

Emerging technologies have the potential to address the barriers of current forms of treatment. The development and ubiquity of the Internet, bolstered by the subsequent advent of smart devices, cultivated a global environment of hyper-connectivity and increased access to information. Increasingly, we see mobile health (mHealth) interventions aiming to address a myriad of mental health and substance use issues by leveraging the increased functionality of mobile devices and mobile phones with app capabilities (smartphones). A significant shift has occurred in the mHealth field, moving primarily from telephone calls and short message service (SMS) interventions to a wide scope of mobile apps with multiple functions and features [14]. These robust mHealth interventions can now provide user-friendly and accessible tools such as evidence-based information and guidelines, personalized reminders, self-assessment tools, goal setting and tracking tools, online resources (eg, webpages, discussion groups, etc), and, with user’s consent, geolocation services to alert users of “high risk” locations [15]. The use of these mobile apps have a significant role in overcoming barriers that lead to attrition of patient participation in traditional AUD treatment programs. Despite this potential, the effectiveness of AUD apps is not well understood. A recent systematic review [16] on mobile technology-based interventions for adult users of alcohol found studies reporting positive effects (eg, reduction in drinking and readiness to change); however, the review focused on all mobile technology interventions including Web- and SMS-based interventions. This proposed systematic review specifically aims to understand the impact of app-based interventions on individuals endorsing symptoms for AUD.

## Methods

The purpose of this systematic review is to assess the effectiveness of app-based interventions in reducing alcohol consumption for individuals with AUD and to understand the client experience. Using a population, intervention, comparator, outcomes (PICO) [17] method to frame the research question, this review aims to determine the effects of mobile apps (intervention) for individuals with AUD (population) compared to a baseline or a control group (comparator, if available) on reducing alcohol consumption (outcome). A secondary objective is to identify other effects related to supporting self-management behaviors or behavior change (eg, self-efficacy, patient activation, patient experience, attrition, sustained use, etc). This systematic review protocol was written using the PRISMA-P statement [17] as a guide and was registered with the PROSPERO database of systematic reviews (#CRD42016049957).

### Search Strategy

The search strategy will be developed in consultation with a health information specialist (SL) at the Centre for Addiction and Mental Health in Toronto, Canada. The search strategy will be first developed for Medline and then translated to query 5 other databases. A mHealth search hedge comprised of the Medline MeSH terms (ie, “Computers/Handheld”, “Mobile Applications/”, “Cell Phones”, “Text Messaging”) and Boolean objects for mobile phones (eg, “(cellphone app* or phone app* or smartphone app*).mp ”) will serve as the foundation of the search. The query will be added to a search for alcohol-related disorder (ie, “alcohol drinking/ or binge drinking/” or “alcohol-related disorders/ or alcoholic intoxication/ or alcoholism/” or “binge drink*.mp) using the “AND” operator. The search will be restricted from 2007 to January 14th, 2016. The year 2007 was selected because this marked the introduction of the Apple iPhone and the popularization of the mobile phone app ecosystem [14]. A second search was conducted during the week of July 15th, 2016 to capture more recent citations.

The search will be applied to 5 health sciences databases (Medline, MEDLINE-IP, PsycINFO, EMBASE, CINAHL). Interdisciplinary databases (Scopus and Web of Science) will also be queried to capture publications from other disciplines such as computer and information systems sciences. If the search results include conference abstracts and proceedings, a search for follow-up articles to the conference abstracts will be conducted on Google Scholar. Lastly, the reference lists of relevant systematic reviews will be hand-combed to identify articles that may be of relevance to this review.

The results from the search will be exported in “.ris” format to import results into a Mendeley citation manager where duplicate citations are de-duplicated using the citation manager. Results from the databases will also be exported as “.xls” files to use as a foundation to develop the screening forms. Additional results from the hand searching and follow-up will be added to Mendeley and documented in a separate spreadsheet. All spreadsheets will be collated into one workbook and developed into a screening form for selecting articles.

### Selection Criteria

Two rounds of screening will be conducted by the two reviewers (AS and YX) to determine the eligibility of a citation or article. Every article will be rated independently by both reviewers and documented on a screening spreadsheet. At the end of each round, the ratings will be compared and discrepancies resolved first by consensus between the reviewers or by a third reviewer (NS) if consensus cannot be reached.

Articles will be first assessed for relevance based on a scan of the title and abstract. Articles meeting all of the inclusion criteria (Textbox 1) will be labeled as potentially relevant and qualified for full-text review. Articles will be excluded if they meet any of the exclusion criteria (Textbox 1).

**Inclusion and exclusion criteria.**

Criteria

- Inclusion criteria
  - a quantitative primary study
  - focused on supporting individuals with alcohol use disorder (AUD), alcohol abuse, or alcohol dependence [4]
  - a consumer-facing app (ie, not for clinician support)
- Exclusion criteria
  - the mobile intervention is only SMS-based, Web-based, or uses interactive voice response (IVR)
  - the mobile app is used as a screening tool rather than an intervention
  - the study is not alcohol specific (ie, addresses poly-substance use and/or addictive behaviors such as gambling, sex, eating, etc)

The full-text review will be used to corroborate the eligibility of “potentially relevant” and hand-searched articles. To be included, the full-text must be in English and the study must be a formal empirical evaluation of the clinical outcomes of using the intervention, where experimental and quasi-experimental designs were included. Formative evaluations, such as usability studies or needs assessments, were excluded.

### Data Extraction

A standardized data extraction form developed by the research team will be used to extract data. The form will be pre-piloted by the two reviewers to ensure there is a common understanding of the categories and formatting for extraction. The extracted information will follow the PICO framework. The study details, population, intervention, and comparator data will be extracted as reported by the article under their respective headings and subheadings. The intervention subheadings will focus on the app design elements (ie, functions, content, and theoretical foundation) and the implementation of the app for the study (ie, dosage and/or duration and human interaction). App functions will include psychoeducation (ie, information), medical assessment, symptom management, supportive resources (ie, peer and/or provider support), therapeutic treatment (ie, behavior change support), and other [18-20]. The other category will be for instances where the function does not fit under the prescribed categories. A list of headings, subheadings, descriptions, and examples of extraction format is provided in Table 1.

**Table 1:** Extraction form elaboration table.

PICO^a^ category		Instruction and/or definition
Study details		
	Study year	When was this study conducted?
	Country	Where is the study from (ie, country)?
	Study design	What type of study was it, as described by article?
	Follow-up	How long was the intervention? And additional follow-up?
	Outcome measures	What did the authors identify as their outcome measures?
	Use of theory	Did the evaluation use theory?
Population		
	Descriptor	What was the study population, as described by the article?
	Inclusion criteria	Are there specific symptoms or patient characteristics?
	Age	What was the average age (or majority age range for majority)?
	Sampling strategy	What was the sampling strategy (ie, randomized, convenience)?
	Sampling frame	Where were participants sampled from?
	Sample size	Was the ratio of completion to recruited reported?
Intervention		
	Intervention name	What is the name of the app?
	App functions description	What does the app do (ie, psychoeducation, medical assessment, symptom management, supportive resources, therapeutic treatment, and “other”)?
	App content	Is there an information component and what topics are provided?
	Theoretical foundation	What theory or model was used for the intervention?
	Duration/dose	Was there prescribed use? If so, what was it, how often, and for how long?
	Human interaction	Did the intervention include interaction with peers or healthcare providers?
Outcomes		
	Primary outcome	What were the primary outcome(s) results, categorized as an “outcome evaluation” or “assessment of drinking behavior”?
	Secondary outcomes	What were the secondary outcome(s) results?

^a^PICO: population, intervention, comparator, outcomes.

The primary outcome of interest is the reduction in alcohol consumption. Based on the domains outlined by the National Institute of Alcohol Abuse and Alcoholism (NIAAA) [16], these outcomes are classified under the “outcome evaluation” and “assessment of drinking behavior” domains. The outcome evaluation domain pertains to the measures that are the end results of treatment, such as the Drinking Problems Index (DPI), the Alcohol Timeline Follow Back (TLFB), and the Addiction Severity Index (ASI). The assessment of drinking behavior domain consists of measures which assess the quantity, frequency, intensity, and pattern of alcohol consumption, for example, the Drinking Self-Monitoring Log (DSML). The secondary outcomes of interest will include measures under the NIAAA “treatment and process assessment” domain. The measures under this domain focus on understanding the process of treatment such as treatment atmosphere, degree of treatment structure, and the immediate goals or proximal outcomes of treatment, for example, the Treatment Services Review (TSR). Measures related to client experience (eg, self-efficacy, patient activation, and patient experience) are also of interest. In addition, participant attrition is a secondary outcome of interest; however, this outcome will be extracted under the population domain.

### Data Analysis

A narrative synthesis of all included articles will be outlined in tables and will include study details, sample characteristics, description of app-based interventions, and outcomes. A meta-analysis will be performed if feasible. Depending on outcomes reported, this review will calculate and report pooled risk ratios or standardized mean differences. Fowler et al [16] found many of the studies in their review did not provide the appropriate data for meta-analysis and that there was too much heterogeneity in populations, interventions, and outcome measures. Based on this observation, effect sizes will be calculated for articles that did not report them using the reported data (ie, test statistics or probability values) to calculate the appropriate metric [21]. Furthermore, a random effects model will be performed since it is anticipated that the included studies may be heterogeneous. The results will be assessed using the evolution of heterogeneity (*I*^2^) statistic [22,23]. A sensitivity analysis will be conducted based on study quality and study design to investigate the source of heterogeneity. Furthermore, a subgroup analysis to explore heterogeneity of the estimated effect sizes will be conducted based on the following characteristics: study quality, severity of AUD in participants, length of intervention, type of control condition, attrition rates, intervention characteristics (automated versus health care provider involvement, theoretical or atheoretical, dosage/duration), and patient experiences (ie, satisfaction, activation, and self-efficacy). Evidence of publication bias will be assessed through funnel plots.

### Quality Assessment

Two reviewers will independently assess the risk of bias in the included studies using the Scottish Intercollegiate Guidelines Network (SIGN) 50 critical appraisal checklists for randomized controlled trials (RCTs) and observational studies [24]. The SIGN 50 tools were selected following recommendations made by the Canadian Agency for Drugs and Technology in Health (CADTH) [25] and were based on a systematic review followed by expert and stakeholder consultation/vetting processes on the quality of the identified quality appraisal tools. The assessments will be compared and disagreements will be resolved by consensus or third reviewer if necessary.

## Results

**Figure 1:**
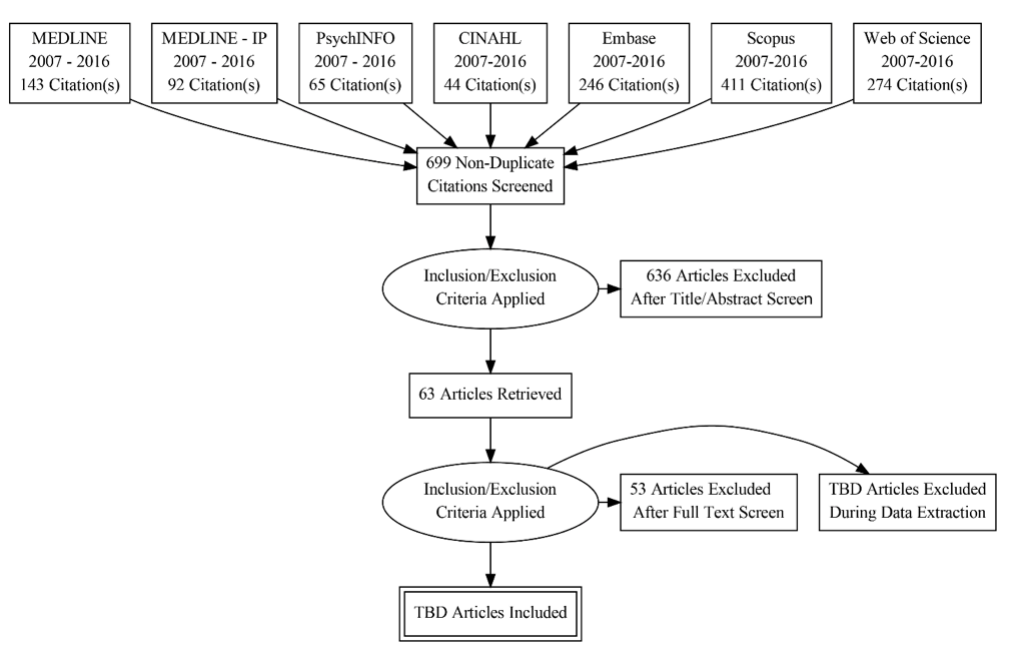
Preliminary PRISMA flow diagram (progress to date).

The initial search yielded 1076 articles of which 570 (52.97%, 570/1076) were unique citations. The second search added an additional 129 (64.8%, 129/199) unique citations. After applying the inclusion and exclusion criteria to the title and abstracts, 63 (9.0%, 63/699) citations were assessed as potentially relevant for full-text review. The hand-searching yielded 6 citations to be included in the full-text review. To date, the reviewers have included 10 articles and will begin data extraction and quality assessments. The progress to date is outlined in Figure 1. This review is projected to be completed in the summer of 2017.

## Discussion

### Principal Findings

There is great potential for apps to support individuals with AUD. However, the efficacy or effectiveness of these apps, or apps in general, is not well understood because the research has yet to catch up to the ever-evolving and expanding mHealth landscape. In 2012, a study found 44 of 500 alcohol-related mobile phone apps available in the Apple and Android marketplaces focused on alcohol reduction [26]. A follow-up review [27] found the number of alcohol-reduction apps (n=91) doubled in two years and that 16.4% of the apps identified (n=662) mentioned evidence in their descriptions. While there are no systematic reviews evaluating the effectiveness of mobile apps, reviews found that electronic interventions (e-interventions) for alcohol misuse in general have short-term benefits in reducing alcohol consumption [28-32]. A recent systematic review [16] of mobile-based interventions for adult alcohol users found only 2 studies on the effectiveness of alcohol reduction/cessation apps, as the remaining articles focused on SMS-based interventions. This review will build on the efforts by Fowler et al [16] by focusing on evaluations of app-based interventions for AUD by expanding the search strategy and including a wider range of study designs.

### Conclusion

This systematic review will be the first to determine the effectiveness of app-based interventions to reduce alcohol consumption for individuals with AUD. While the primary focus will be clinical outcomes, this study will also seek to understand whether using the app will have any positive psychological outcomes in the form of empowerment or self-efficacy and whether these apps provide the support needed to sustain use. Engagement with the app is important if behavior change is to occur, especially with many digital interventions experiencing high rates of attrition [27,33]. Understanding these secondary factors will provide insights on the behavioral mechanism required to reduce alcohol consumption and whether there are gaps between app concept, delivery, and translation into behavior change and alcohol use reduction [33].

## Abbreviations

AUD: alcohol use disorder
mHealth: mobile health
NIAAA: National Institute of Alcohol Abuse and Alcoholism
PICO: population, intervention, comparator, outcomes
SIGN: Scottish Intercollegiate Guidelines Network
